# Supplementary material for: Succession Analysis of Gut Microbiota Structure of Participants from Long-Lived Families in Hechi, Guangxi, China
Source: Microorganisms. 2021 Dec 7;9(12):2524. doi: 10.3390/microorganisms9122524 (PMC8703768; doi:10.3390/microorganisms9122524)
Supplement: Supplementary file 1 [file microorganisms-09-02524-s001.zip › microorganisms-1470970-Supplementary.pdf]

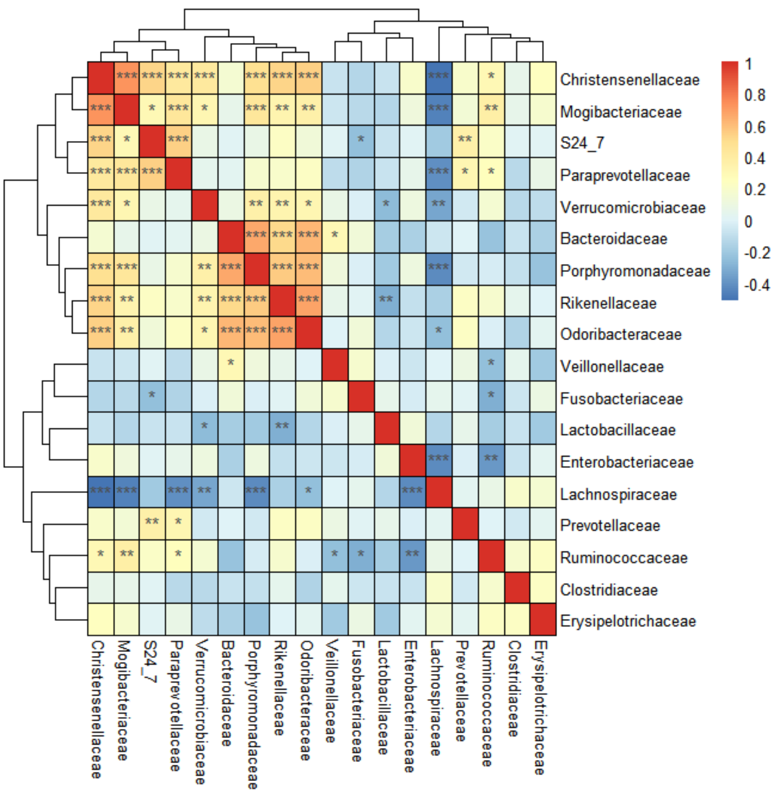

Figure S1. Heat map of the Spearman rank correlations between the family-level phylotypes. Red indicates a positive correlation and blue indicates a negative correlation; * p-value<0.05; ** p-value<0.01; *** p-value<0.001. N= 69 participants in long-lived families

Table S1. Detailed information of all participants.

**Participant No.**	Age	Gender	BMI (kg/m²)	Group	**Participant No.**	Age	Gender	BMI (kg/m²)	Group
1	46	male	21.3	LEA	50	102	female	25.3	LCN
2	58	female	25.7	LEA	51	111	female	17.7	LCN
3	56	female	18.0	LEA	52	100	female	18.6	LCN
4	52	female	24.7	LEA	53	103	female	16.8	LCN
5	40	female	20.3	LEA	54	104	female	21.3	LCN
6	51	male	28.7	LEA	55	106	female	20.1	LCN
7	56	female	20.3	LEA	56	107	female	22.8	LCN
8	50	male	24.2	LEA	57	108	female	16.1	LCN
9	38	male	24.5	LEA	58	100	male	17.8	LCN
10	55	male	19.3	LEA	59	100	male	17.1	LCN
11	56	female	24.6	LEA	60	101	female	18.6	LCN
12	52	female	26.6	LEA	61	101	female	19.1	LCN
13	49	male	26.7	LEA	62	102	female	19.2	LCN
14	52	male	25.0	LEA	63	102	female	19.7	LCN
15	51	male	22.7	LEA	64	102	female	17.7	LCN
16	44	female	18.7	LEA	65	102	female	19.4	LCN
17	69	male	29.0	LEA	66	104	female	20.0	LCN
18	61	female	22.0	LEA	67	107	female	19.2	LCN
19	66	male	27.0	LEA	68	108	female	18.4	LCN
20	64	female	18.1	LEA	69	118	female	22.8	LCN
21	61	male	19.5	LEA	70	66	male	21.1	UEA
22	72	female	19.8	LEA	71	61	female	20.4	UEA
23	76	female	24.1	LEA	72	61	male	24.8	UEA
24	65	male	20.3	LEA	73	65	male	21.8	UEA
25	62	female	18.7	LEA	74	65	female	25.3	UEA
26	60	male	18.4	LEA	75	70	male	21.6	UEA
27	66	male	24.6	LEA	76	82	female	27.9	UEA
28	60	male	17.3	LEA	77	80	male	29.3	UEA
29	63	male	23.2	LEA	78	79	female	18.2	UEA
30	80	female	15.4	LEA	79	63	male	23.0	UEA
31	60	male	18.6	LEA	80	72	female	25.0	UEA
32	91	male	20.0	LCN	81	64	male	25.7	UEA
33	92	female	20.4	LCN	82	70	female	20.7	UEA
34	92	female	20.1	LCN	83	71	male	25.6	UEA
35	93	male	17.6	LCN	84	53	male	23.6	UEA
36	94	male	22.0	LCN	85	54	female	25.6	UEA
37	98	female	17.4	LCN	86	55	male	21.6	UEA
38	91	female	22.6	LCN	87	59	female	26.0	UEA
39	91	female	19.6	LCN	88	59	female	21.5	UEA
40	91	female	19.2	LCN	89	56	male	25.1	UEA
41	92	female	20.5	LCN	90	54	female	21.8	UEA
42	90	female	12.4	LCN	91	28	female	21.2	UEA
43	91	female	18.1	LCN	92	56	female	17.9	UEA
44	92	female	14.7	LCN	93	31	female	20.3	UEA
45	93	female	17.3	LCN	94	33	male	24.0	UEA
46	93	female	18.1	LCN	95	32	female	18.8	UEA
47	93	female	19.8	LCN	96	28	female	18.5	UEA
48	95	female	18.4	LCN	97	56	male	21.4	UEA
49	96	female	19.9	LCN	98	56	male	19.6	UEA

Table S2. Metabolic pathways with significant differences between the long-lived elderly group and the offspring group.

KEGG pathway	Class	Mean Relative abundance（%）		*p*-value
		LCN	LEA	
Pentose phosphate pathway	Metabolism; Carbohydrate metabolism	1.73	1.78	0.003
Propanoate metabolism	Metabolism; Carbohydrate metabolism	0.72	0.67	0.004
Starch and sucrose metabolism	Metabolism; Carbohydrate metabolism	1.17	1.26	0.002
Butanoate metabolism	Metabolism; Carbohydrate metabolism	0.78	0.73	0.043
Citrate cycle (TCA cycle)	Metabolism; Carbohydrate metabolism	0.93	0.83	0.002
Galactose metabolism	Metabolism; Carbohydrate metabolism	1.10	1.20	0.002
Porphyrin and chlorophyll metabolism	Metabolism; Metabolism of cofactors and vitamins	0.74	0.79	0.002
Thiamine metabolism	Metabolism; Metabolism of cofactors and vitamins	1.65	1.77	0.008
Ubiquinone and other terpenoid-quinone biosynthesis	Metabolism; Metabolism of cofactors and vitamins	0.43	0.32	0.037
Lipoic acid metabolism	Metabolism; Metabolism of cofactors and vitamins	0.81	0.54	0.001
Primary bile acid biosynthesis	Metabolism; Lipid metabolism	0.09	0.11	0.025
Secondary bile acid biosynthesis	Metabolism; Lipid metabolism	0.81	0.99	0.025
Sphingolipid metabolism	Metabolism; Lipid metabolism	0.40	0.44	0.036
Glycerolipid metabolism	Metabolism; Lipid metabolism	0.54	0.58	0.031
Valine, leucine and isoleucine degradation	Metabolism; Amino acid metabolism	0.35	0.30	0.002
Tryptophan metabolism	Metabolism; Amino acid metabolism	0.20	0.18	0.021
Glycine, serine and threonine metabolism	Metabolism; Amino acid metabolism	1.12	1.09	0.007
Lysine degradation	Metabolism; Amino acid metabolism	0.20	0.18	0.036
Streptomycin biosynthesis	Metabolism; Biosynthesis of other secondary metabolites	1.64	1.74	0.016
Taurine and hypotaurine metabolism	Metabolism; Metabolism of other amino acids	0.69	0.64	0.009
Biosynthesis of vancomycin group antibiotics	Metabolism; Metabolism of terpenoids and polyketides	2.07	2.32	0.002
Carbon fixation pathways in prokaryotes	Metabolism; Energy metabolism	1.08	1.00	0.001
Dioxin degradation	Metabolism; Xenobiotics biodegradation and metabolism	0.28	0.32	0.043
Glycosaminoglycan degradation	Metabolism; Glycan biosynthesis and metabolism	0.34	0.25	0.030
Sulfur relay system	Genetic Information Processing; Folding, sorting, and degradation	1.20	1.27	0.011
Base excision repair	Genetic Information Processing; Replication and repair	0.92	0.88	0.006
Cell cycle - Caulobacter	Cellular Processes; Cell growth and death	1.40	1.47	0.026

Table S3. Correlations between age and gut microbiota at family and genus level.

Bacterial Taxa	Age	
	Pearson correlation coefficient	*p*-value
Porphyromonadaceae	0.29*	0.017
Lactobacillaceae	-0.02	0.870
Christensenellaceae	0.12	0.334
S24-7	0.066	0.598
Paraprevotellaceae	-0.139	0.263
Rikenellaceae	0.251*	0.041
Clostridiaceae	-0.053	0.669
Erysipelotrichaceae	-0.174	0.158
Veillonellaceae	-0.246*	0.045
Fusobacteriaceae	-0.07	0.574
Verrucomicrobiaceae	0.131	0.290
Prevotellaceae	-0.415***	0.000
Bacteroidaceae	0.141	0.257
Lachnospiraceae	-0.477	0.000
Enterobacteriaceae	0.300*	0.014
Ruminococcaceae	0.035	0.779
Mogibacteriaceae	0.336**	0.005
Odoribacteraceae	0.287*	0.018
Parabacteroides	0.241*	0.049
Lactobacillus	-0.02	0.87
Citrobacter	-0.137	0.269
Phascolarctobacterium	-0.269	0.028
Cetobacterium	0.027	0.831
Dorea	-0.172	0.164
Fusobacterium	-0.127	0.304
Lachnospira	-0.114	0.357
Coprococcus	-0.096	0.44
Clostridium	0.039	0.752
Klebsiella	0.059	0.636
Akkermansia	0.131	0.29
Blautia	-0.331**	0.006
Oscillospira	0.227	0.065
Prevotella	-0.31*	0.011
Gemmiger	-0.017	0.888
Ruminococcus	-0.006	0.959
Roseburia	-0.418***	0.000
Faecalibacterium	-0.191	0.121
Bacteroides	0.141	0.257
Escherichia	0.304	0.012
Bifidobacterium	-0.061	0.624
Christensenella	0.037	0.766

The results were adjusted by gender and BMI * p-value < 0.05; ** p-value < 0.01; *** p-value < 0.001.

Table S4. Correlations between BMI and gut microbiota at family level.

Bacterial Taxa	BMI	
	Spearman correlation coefficient	*p*-value
Porphyromonadaceae	-.255*	0.034
Rikenellaceae	-.314***	0.009
Christensenellaceae	-0.206	0.089
Verrucomicrobiaceae	-.275*	0.022
Mogibacteriaceae	-0.13	0.287
Odoribacteraceae	-.285*	0.018
Lactobacillaceae	0.191	0.115
S24-7	0.087	0.479
Paraprevotellaceae	0.141	0.249
Clostridiaceae	-0.166	0.173
Erysipelotrichaceae	0.014	0.907
Veillonellaceae	-0.17	0.163
Fusobacteriaceae	-0.148	0.224
Prevotellaceae	-0.074	0.548
Bacteroidaceae	-0.158	0.196
Lachnospiraceae	0.147	0.229
Enterobacteriaceae	0.016	0.897
Ruminococcaceae	0.071	0.56

* p-value < 0.05; ** p-value < 0.01; *** p-value < 0.001.
